# Neural Correlates of Belief-Bias Reasoning as Predictors of Critical Thinking: Evidence from an fNIRS Study

**DOI:** 10.3390/jintelligence13090106

**Published:** 2025-08-24

**Authors:** Juanjuan Ma, Wenyu Lv, Xuezhu Ren

**Affiliations:** 1 School of Education, Huazhong University of Science & Technology, Hongshan District, Wuhan 430074, Chinam202375415@hust.edu.cn (W.L.); 2 Department of Pulmonary and Critical Care Medicine, The Sixth Hospital of Wuhan, Affiliated Hospital of Jianghan University, Jiangan District, Wuhan 430019, China

**Keywords:** belief-bias reasoning, critical thought, metacognition, analytic thinking

## Abstract

This study examined the neural characteristics of belief-bias reasoning in order to reveal the neurocognitive basis of critical thinking. Functional near-infrared spectroscopy was utilized to capture the real-time brain hemodynamic activity of 74 college students while they performed a belief-bias syllogistic reasoning task. Values of oxy-hemoglobin (oxy-Hb) and deoxy-hemoglobin (deoxy-Hb) in regions of interest were analyzed in relation to critical thinking skills assessed by established tests. The results reveal significant activation in both the opercular part of the right IFC and the left DLPFC when participants encountered situations where their prior beliefs contradicted logical validity during the completion of the belief-bias syllogistic reasoning task. Crucially, individuals with lower levels of critical thinking skills demonstrated heightened activation in the opercular part of the right IFC compared to those with higher levels of critical thinking skills. Furthermore, variations in hemodynamics, quantified by oxy-Hb and deoxy-Hb concentration values (area under the activity curve as absolute value), during the execution of belief-bias reasoning tasks accounted for a substantial proportion of the variability in critical thinking skills. Additionally, the hemodynamic data to a large extent explained the connection between belief-bias reasoning and critical thinking. These results provide a neural explanation for the relationship between belief-bias reasoning and critical thinking, and advance theoretical models of critical thinking by illuminating the brain’s mechanisms engaged in unbiased reasoning and metacognition.

## 1. Introduction

Critical thinking has been described as rational and goal-oriented thinking that is vital for effective problem solving, drawing inferences, and making informed decisions (Halpern 2014; Halpern and Dunn 2021). It involves objectively assessing information and evidence, free from the influence of prior beliefs (Bensley 2023; Van Peppen et al. 2021; West et al. 2008). Many classic models conceptualize critical thinking as a multidimensional construct that encompasses skills, dispositions, and other factors (e.g., Paul and Elder 2006; Saiz and Rivas 2008). For example, Saiz and Rivas (2008) consider the dispositional dimension as essential for distinguishing a competent thinker from a merely technical one. Critical thinking is often defined as a metacognitive process involving various skills and dispositions, enacted through self-regulatory reflective judgment (Bensley 2020; Dwyer et al. 2014; Halpern 2014; Rivas et al. 2022). In an era marked by the rapid rise of generative artificial intelligence (AI) and as reliance on AI-generated information grows across education, communication, and decision-making, critical thinking has become essential for evaluating sources, detecting biases, and resisting misinformation. Recognizing this need, a growing number of nations have emphasized the significance of developing critical thinking skills in students (e.g., National Education Association 2024; UNESCO 2024) and educators widely acknowledge this as a key objective of education. To achieve this goal, however, a comprehensive understanding of individual differences in critical thinking and their neural mechanisms is required. This study employs functional near-infrared spectroscopy (fNIRS) to investigate hemodynamic changes during belief-bias syllogistic reasoning in an attempt to reveal the neurocognitive basis of critical thinking.

### 1.1. Critical Thinking and Belief-Bias Reasoning

Theoretical and empirical research suggests that addressing belief bias during reasoning is an essential aspect of critical thinking (Bensley 2023; Evans et al. 2022; Stanovich and Toplak 2019; Van Peppen et al. 2021; West et al. 2008). People often accept a conclusion if it aligns with their prior beliefs, regardless of its logical validity. Belief bias occurs when a person’s prior beliefs overshadow the logical validity of reasoning, leading them to accept or reject a conclusion based on believability rather than its logical soundness. The ability to set aside one’s prior knowledge and consider premises objectively is seen as a hallmark of primary critical thinking skills. The critical thinking literature places great importance on the ability to overcome belief bias during reasoning (e.g., Evans et al. 2022; Halpern 2014; West et al. 2008). The belief-bias syllogism is a common paradigm for measuring belief bias during reasoning, which involves non-conflict trials where the conclusion of a syllogism is consistent with prior beliefs, and conflict trials where the conclusion is inconsistent with beliefs. Belief bias can facilitate logical reasoning in non-conflict trials but obstruct logical reasoning in conflict trials (Evans and Stanovich 2013).

The relevance of belief bias during reasoning to critical thinking can be traced to the dual process theory of reasoning, which proposes two types of processes: type 1 processing and type 2 processing (Evans and Stanovich 2013). Type 1 processing, also called heuristic thinking, relies on prior knowledge and beliefs to solve problems, and if the conclusion aligns with or conflicts with previous knowledge, it will facilitate or obstruct the conclusion regardless of its logical validity. Type 2 processing relies heavily on cognitive resources such as working memory and executive functions, and only becomes available when a conflict between heuristic and analytical responses is detected. It then serves to suppress the dominant heuristic response (Evans and Stanovich 2013). Halpern (2014) further proposes that type 2 processing is a requirement for critical thinking and that it always results in critical thinking. Bensley (2020) concurs that a key function of critical thinking is to resist default heuristic responses, including those that are belief-biased.

Reflecting on one’s beliefs and correcting potential biases is also considered a form of metacognition, vital to critical thinking (Bensley 2020; Dwyer et al. 2014; Halpern 2014; Rivas et al. 2022). Metacognition, encompassing knowledge about cognition and self-regulation, serves as a bridge connecting belief bias during reasoning and critical thinking (Bensley 2020; Guamanga et al. 2025; Halpern 2014). In particular, self-regulation underlines the importance of metacognitive processes, including executive functions, in resolving conflicts between logical and belief-based processing (De Neys 2018). Halpern (2014) argues that critical thinkers assess thought processes and their outcomes, thus establishing a connection between metacognition and critical thinking. Similarly, Lau (2015) emphasizes that critical thinking emerges when metacognition is effectively employed to enhance the accuracy of reasoning. The contemporary framework for critical thinking also emphasizes the pivotal role of metacognition in critical thinking (Dwyer et al. 2014). Essentially, from a theoretical perspective, metacognition strengthens the link between belief-bias reasoning and critical thinking.

Behavioral research also supports the close association between critical thinking and the ability to inhibit belief bias during reasoning (e.g., Van Peppen et al. 2021; West et al. 2008). For example, West et al. (2008) found that university students’ dispositions toward critical thinking were significantly correlated with their ability to inhibit belief bias during reasoning. There is also research showing that providing explicit instructions to decouple prior beliefs in reasoning improved students’ critical thinking skills (Heijltjes et al. 2014; Van Peppen et al. 2021), suggesting a link between belief-bias reasoning and critical thinking. Moreover, the ability to suppress bias caused by prior beliefs is widely recognized as an important component of critical thinking. While not all critical thinking assessments measure this ability directly, several widely used tests, such as the Watson–Glaser Critical Thinking Appraisal, the Cornell Critical Thinking Test, and the California Critical Thinking Skills Test, include items that indirectly tap into belief-bias suppression through tasks requiring logical evaluation of arguments.

### 1.2. Brain Activity Associated with Belief-Bias Syllogistic Reasoning

Though theoretical and behavioral research suggests that the ability to inhibit belief-bias during reasoning is a crucial function of critical thinking, neurocognitive evidence in support of this claim is scarce. Most existing research has focused on the neural characteristics of belief-bias syllogistic reasoning. One line of research has used event-related potentials (ERPs) to examine the temporal aspects of belief-bias syllogistic reasoning, showing that conflict syllogisms elicit larger centro-parietal N200 and frontal P300 responses compared to non-conflict ones (Bago et al. 2018; Banks and Hope 2014). Another line of research, which the current research takes, employed brain imaging techniques such as functional magnetic resonance imaging (fMRI) and fNIRS to investigate the brain regions involved in belief-bias syllogistic reasoning. Research has found that the right inferior prefrontal cortex (IFC) and the left dorsolateral prefrontal cortex (DLPFC) were activated during belief-bias syllogistic reasoning (Dubreuil-Vall et al. 2019; Goel and Dolan 2003; Tsujii and Watanabe 2009).

Goel and Dolan (2003) were among the first to use the fMRI to examine the effect of prior beliefs on syllogistic reasoning. They found that the right IFC was activated when subjects successfully suppressed belief bias during syllogistic reasoning. Conversely, when belief-bias took precedence over logical reasoning, the ventral medial prefrontal cortex was activated, which is implicated in affective processing. They also found that the left DLPFC was recruited during belief-bias syllogistic reasoning. The left DLPFC is associated with conflict monitoring, while the right IFC is responsible for modulating attention monitoring and inhibiting prepotent responses (Dubreuil-Vall et al. 2019; Goel and Dolan 2003; Tsujii et al. 2010), both of which are critical for type 2 processing (Aron et al. 2014).

The role of the right IFC in belief-bias syllogistic reasoning has also been examined by using fNIRS. Tsujii and Watanabe (2009) used fNIRS to study the impact of a secondary task on the IFC activity during belief-bias syllogistic reasoning. They measured changes in oxyhemoglobin (oxy-Hb) and deoxyhemoglobin (deoxy-Hb) concentration values in the left and right IFC. Their results showed that a high-load secondary task impaired participants’ performance on conflict reasoning and led to decreased activation in the right IFC, while there was no decrease in left IFC activity. The authors explain that the high-load secondary task consumed time and attention, leaving insufficient time for participants to initiate type 2 processing, which resulted in decreases in the right IFC activity. This result highlights the pivotal role of right IFC activity in initiating type 2 processing and underscores the distinction between the fast type 1 and slow type 2 processing. Their follow-up work (e.g., Tsujii et al. 2010) focusing on age differences in IFC activity during belief-bias syllogistic reasoning also highlights the role of the right IFC in inhibiting default heuristic type 1 processing.

### 1.3. The Present Study

As stated above, both theoretical and behavioral research suggests that the ability to overcome belief bias during reasoning is a fundamental component of critical thinking. However, there is currently a lack of neural evidence to validate the theoretical connection between belief-bias reasoning and critical thinking. Although neurocognitive research has shown that inhibiting belief bias during reasoning is linked to activity in the right inferior frontal cortex (IFC) and the dorsolateral prefrontal cortex (DLPFC), it remains unclear whether and to what extent this hemodynamic brain activity can elucidate the relationship between belief-bias reasoning and critical thinking. The current study was undertaken to investigate the hemodynamic response related to brain activation during belief-bias syllogistic reasoning and its correlation with critical thinking. We explored potential differences in hemodynamic response between individuals with high and low levels of critical thinking. In particular, we sought to determine the extent to which brain activity during belief-bias reasoning predicts variations in critical thinking.

To accomplish the research objectives, we employed fNIRS to investigate brain activity during belief-bias syllogistic reasoning. fNIRS records cortical hemodynamic responses by assessing the attenuation of near-infrared light as it passes through tissue, detecting changes in oxy- and deoxy-hemoglobin concentrations at wavelengths of around 650 and 1000 nm (Scholkmann et al. 2013). These changes, originating from regional cortical activation, signify variations in cerebral venous blood flow in response to brain activity. Significantly, fNIRS offers superior spatial resolution when compared to EEG and improved temporal resolution compared to MRI. It also boasts non-invasiveness, portability, resistance to bodily movements, and is suitable for probing neural mechanisms in psychological research (Brigadoi et al. 2013).

We recorded participants’ fNIRS data when they completed a belief-bias syllogistic reasoning task. Data on participants’ critical thinking skills were also collected. We compared the hemodynamic activity between individuals with high and low levels of critical thinking abilities. Furthermore, we explored the extent to which the hemodynamic data acquired during belief-bias syllogistic reasoning could predict variations in critical thinking abilities and clarified how these data enhanced our comprehension of the association between belief-bias reasoning and critical thinking.

## 2. Method

### 2.1. Participants

We recruited 78 college students from two universities through advertising. All participants were right-handed, healthy, and had normal or corrected-to-normal vision. One student’s data were excluded from analyses because they showed very low accuracy (less than 25%) in the belief-bias syllogistic reasoning task. The fNIRS data of another three participants were excluded due to the removal of channels exceeding 30%, resulting in a final sample of 74 participants (50 males) with ages ranging from 18 to 22 years (*M* = 19.47, *SD* = 0.74). The study was approved by the Human Subjects Review Committee of the Huazhong University of Science and Technology. All participants provided written informed consent, which described the study purpose, procedures, and their right to free withdrawal. Participants were financially compensated for their participation.

### 2.2. Experimental Task of Belief-Bias Syllogistic Reasoning and FNIRs Data Acquisition

The experimental task was adapted from the belief-bias syllogistic reasoning paradigm, which measures individuals’ ability to evaluate evidence and arguments during reasoning regardless of prior beliefs (Markovits and Nantel 1989). We manipulated the logical validity (valid vs. invalid) and the believability (believable vs. unbelievable) of conclusions in each syllogism. Table S1 presents that the combination of logical validity and conclusion believability yielded two types of trials: conflict trials (valid-unbelievable, invalid-believable) associated with critical thinking, and non-conflict trials (valid-believable, invalid-unbelievable) related to heuristic thinking. Each syllogism consisted of a major premise, a minor premise, and a conclusion, with the length of the premises or conclusion ranging from 4 to 11 Chinese characters. The Chinese character counts of the conflict and non-conflict trials were carefully balanced to ensure that variations in length were equivalent across the two different trial types. To ensure the enduring impact of belief bias on logical judgments, the premises and conclusions of each syllogism were simultaneously presented. The task consisted of 80 trials, with 8 trials being randomly assigned to practice blocks and not subjected to analysis.

**Figure 1:**
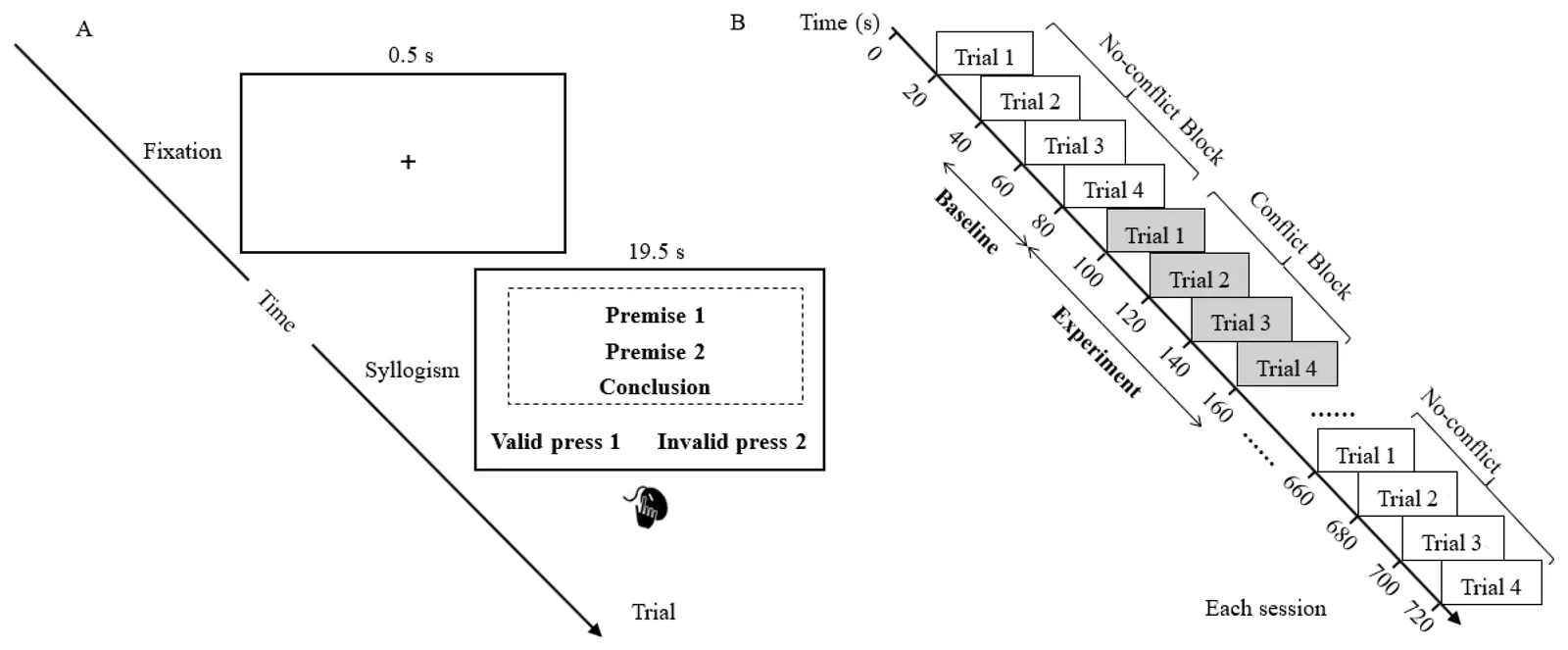
Procedure of the experimental task of belief-bias syllogistic reasoning. Note. Panel (**A**): Trial sequence of the belief-bias syllogistic reasoning task. Panel (**B**): Two types of reasoning, conflict (grey square) and non-conflict (white square) blocks, were presented. Each block included four trials. Participants completed two reasoning sessions each, including four conflict blocks interleaved with five non-conflict blocks.

The task was implemented using the E-Prime Professional version 3.0 (Psychology Software Tools, Inc., Sharpsburg, PA, USA). The task sequence is illustrated in Figure 1. In each trial, participants were initially presented with a fixation “+” for 0.5 s, followed by a syllogism consisting of two premises and one conclusion for 19.5 s. During this period, participants were instructed to assume the premises to be true and to evaluate the validity of each syllogism. To minimize head and facial movement and reduce fNIRS-related motion artifacts, participants were required to press the “1” key if they considered the conclusion to be valid or the “2” key if they found it to be invalid, without receiving feedback. This response method was chosen to reduce motion artifacts, which are known to compromise fNIRS signal quality, while ensuring that task performance could be accurately recorded.

This experiment employed a block design. Participants engaged in two reasoning sessions during which their NIRS signals were recorded. They were allowed to take a short break (3–5 min) between the first and second sessions. Each session consisted of nine blocks, with four conflict blocks interleaved with five non-conflict blocks, each containing four trials (see Figure 1B). Each trial was presented for 20 s, and each block lasted 80 s. The conflict blocks were treated as experimental blocks (80 s) while the last 40 s of the non-conflict blocks served as baseline (see Figure 1B). To familiarize participants with the task, they had to complete a practice block, with an accuracy requirement of 60%. To measure the capacity of inhibiting belief bias, the scores were computed based on the accuracy of conflict trials. The internal consistency reliability (Cronbach’s alpha) of the task was 0.72 in this study.

The fNIRS data were collected using a continuous wave system (BS-7000, Wuhan Znion Technology Co., Ltd., Wuhan, China) operating at 690 and 830 nm wavelengths, with a sampling rate of 100 Hz. The head probe consisted of 53 channels, with 16 pairs of emitters and detectors separated by 3 cm and positioned over the prefrontal regions. An investigator positioned the head probe on a participant’s head symmetrically and with the brim approximately 1 cm above the eyebrows. When participants wore the head probe, a calibration phase was conducted prior to fNIRS recording, with a minimum calibration accuracy threshold of 70% set for approval (Vidaurre and Blankertz 2010). During this calibration process, optical pathways could be fine-tuned to accommodate the unique anatomical features of each participant, thereby guaranteeing the precision of data collection. The actual position of the probes was determined using NIRS-SPM based on the 10–20 system (Jurcak et al. 2007) and locations of the probes in the Montreal Neurological Institute stereotaxic coordinate system (Singh et al. 2005) were depicted in Figure S1 in the Supplementary using the BrainNet Viewer. Figure S1 depicts that optode A, positioned in the bottom row of optodes, was located precisely at the midpoint of the frontal pole, as designated by the 10–20 system notation for the position Fpz.

### 2.3. Critical Thinking Skills Tests

To enhance the validity of our critical thinking assessment and to minimize test-specific biases, we utilized two established critical thinking tests or adaptations that assess a range of critical thinking skills.

Chinese critical thinking test (CCTT). Based on the framework of the California Critical Thinking Skills Test (Facione et al. 2002), the CCTT was adapted to suit the cultural and academic backgrounds of Chinese university students (Luo and Yang 2002). The test consisted of 34 items that evaluated five dimensions of critical thinking skills: analysis, evaluation, deduction, inductive reasoning, and inference reasoning. Analysis involves identifying the intended and actual relationships between forms of representation that express beliefs, judgments, experiences, reasons, information, or opinions. Evaluation involves assessing the credibility of claims and the strength of arguments. Deductive reasoning entails the process of making reasoned judgments in logically rigorous contexts. Inductive reasoning encompasses reasoning and judgment in uncertain and ambiguous contexts. Inference reasoning involves identifying and securing the elements necessary to draw reasonable conclusions. The Supplementary Materials provide example items for each of the skills. Each item had four or five response options, with only one being correct, and participants were asked to choose the most appropriate option. The score was the number of correctly answered items.

According to Luo and Yang (2002), the split-half reliability and test-retest reliability of the CCTT were 0.80 and 0.65, respectively. The construct validity of the test is supported by moderate to strong correlations between the scores of the subscales and the total score. Our study found the internal consistency reliability of the test to be 0.70. Furthermore, the CCTT showed a moderately strong correlation (*r* = 0.61, *p* < 0.01) with another critical thinking skills test used in this study (i.e., the critical thinking skills test with heuristics and biases), indicating convergent validity.

Critical thinking skills test with heuristics and bias (CTHB). This test was an adaptation of previous critical thinking skills measures used by Heijltjes et al. (2014) and West et al. (2008). It assesses critical thinking skills by evaluating a person’s ability to avoid heuristics and biases, and it consists of six categories, with an example task included for each in the Supplementary Material: (1) Causal base-rate tasks, measures the tendency to overvalue expert opinion and large sample information, while undervaluing simple probability rules; (2) Wason selection tasks, evaluates a person’s tendency to verify rules rather than falsifying them, as well as their consideration of only lexically matching information; (3) Conjunction fallacy tasks, assess the extent to which people neglect the conjunction rule of probability theory (P(A&B) ≤ P(B)); (4) Covariation detection tasks, evaluates the unequal assessment of information in a 2 × 2 contingency table, based on prior experience and disregard of some presented evidence; (5) Cognitive reflection tests, evaluates people’s tendency to overlook an incorrect advantageous response and their inclination towards further reflection to achieve the correct response; (6) Syllogistic reasoning tasks, assesses the tendency to evaluate the logical validity of conclusions regardless of prior beliefs. The CTHB test consists of 30 items in total, with 4 items for each of the first five categories and 10 items for the syllogistic reasoning tasks. The items are in multiple-choice format with varying numbers of alternatives and there is only one correct answer for each item. The internal consistency of the test, as measured by Cronbach’s alpha, was 0.87 in the study.

### 2.4. Procedure

Participants were individually assessed in a quiet laboratory setting. The critical thinking skills tests were administered using a desktop computer and participants selected their answers by clicking on the response options with a mouse. This method was employed in accordance with standardized computerized testing procedures to ensure consistency, objectivity, and ease of data collection. Prior to commencing the laboratory experiment, each participant was asked to provide demographic information, including age and gender, on a data sheet. The belief-bias syllogistic reasoning task was then administered using the E-Prime software while simultaneously measuring the participants’ brain activity through fNIRS. Participants were instructed to remain relaxed and minimize head movements during the experiment. The entire experiment lasted approximately 30 min for each participant.

### 2.5. Data Analyses

#### 2.5.1. The fNIRS Data Pre-Processing

Data pre-processing was performed using both the Homer 2 (MGH–Martinos Center for Biomedical Imaging, Boston, MA, USA) and the NIRS-SPM (Bio Imaging Signal Processing Lab, Daejeon, Republic of Korea) implemented in MATLAB version R2013b (The MathWorks, Natick, MA, USA). In the Homer2 interface, we first pruned the channels by excluding channels with optical intensities higher than 2.5 or lower than 0.01, which exceeded the detection range of the instrument. To assess the signal-to-noise performance of a data channel, we evaluated the stability of the channel signals by calculating the coefficient of variation (CV) for each channel and eliminating channels with CV > 0.15 (Piper et al. 2013). In the end, approximately 10–20% of data channels were eliminated for each participant.

The optical intensity raw data were then converted to optical density data. Segments of data surrounding the timepoint of motion artifacts were identified and marked if any active data channel exhibited a signal change greater than the standard deviation threshold or amplitude threshold (Scholkmann et al. 2013). These motion artifacts were then corrected by performing a cubic spline correction procedure. The cubic spline correction aimed to reduce noise, remove artifacts, and improve the accuracy of the underlying data representation. All channels underwent low-pass filtering with a 0.01 Hz frequency using Homer 2. This filtering, based on the inverse of the fNIRS instrument’s 100 Hz sampling rate, was used to remove high-frequency noise. Considering the limitations of setting frequency threshold to remove the global trends linked to breathing, cardiac activity, vaso-motion, and experimental factors, we employed the Wavelet MDL detrending algorithm method from NIRS-SPM for high-pass filtering to ensure data quality (Jang et al. 2009). The Wavelet transform efficiently dissects NIRS measurements into distinctive scales representing global trends, hemodynamic signals, and uncorrelated noise components. The optical density data were then converted to the oxy-Hb and deoxy-Hb concentrations using the modified Beer–Lambert law (cf. Wyatt et al. 1986), according to which both the oxy-Hb and deoxy-Hb signals are used for measuring changes in the cerebral blood flow.

#### 2.5.2. The fNIRS Data Analysis

We employed the baseline-corrected block averaging method to examine individual variations in brain activity. This method aligned with our block design that emphasized the hemodynamic changes within experimental blocks relative to the baseline. The baseline-corrected block averaging method is a conventional approach for analyzing fNIRS data, which enhances data quality by reducing baseline drift or noise without making assumptions about signal characteristics (Tak and Ye 2014).

The mean oxy-Hb and deoxy-Hb concentrations were calculated by averaging the oxy-Hb and deoxy-Hb concentrations across blocks for each channel and participant, utilizing the block averaging procedure in Homer 2. We extracted the area under the activity curve as the hemodynamic feature since it has been demonstrated to be more accurate and sensitive than mean values of oxy-Hb and deoxy-Hb for Spatio-temporal feature extraction in cognitive research (Silveira 2021). The average relative values of oxy- and deoxy-Hb across experimental blocks (80 s) minus the baseline (40 s) were calculated, serving as an indicator of the ability to resolve conflicting reasoning (Evans and Stanovich 2013; Tsujii and Watanabe 2009; Tsujii et al. 2010). Notably, the timing of the baseline was determined by conducting multiple pre-experiments and aligned with the experimental design proposed by Tsujii and Watanabe (2009) and Tsujii et al. (2010).

To obtain the *t*-statistic maps based on the 53 channels during belief-bias reasoning, paired-samples *t*-tests were employed to compare the concentration values (area under the activity curve) between experimental and baseline blocks for each channel. The resulting *p*-values were adjusted using the false discovery rate (FDR) correction method (*p* < 0.05) to reduce the potential for Type 1 errors. The FDR correction was carried out using the Benjamini–Yekutieli (BY) procedure (Benjamini and Yekutieli 2001). This procedure provides a robust correction for controlling the FDR while considering a broader range of dependency structures among multiple hypotheses. The FDR corrected *p*-values corresponded to *t*-values, yielding group-level *t*-maps (e.g., Lu et al. 2021). The MNI coordinates and *t*-values of the *t*-maps were converted into files of the IMAG format using xjView (nirs2img.m, http://www.alivelearn.net/xjview, accessed on 15 November 2024). The results of the IMG files were rendered over the 3D brain model using BrainNet Viewer (Lu et al. 2021).

We conducted *t*-tests to examine group differences in the relative oxy- and deoxy-Hb concentration values (area under the activity curve) between participants with high and low critical thinking skills. This analysis specifically focused on two ROIs selected based on prior neuroimaging research, namely the opercular part of the right inferior frontal cortex (IFC, spanning channels 46 and 51) and the left dorsolateral prefrontal cortex (DLPFC, spanning channels 5 and 6) (e.g., Goel 2007; Tsujii et al. 2010). The relative oxy-Hb and deoxy-Hb concentration values within each ROI were averaged across the two channels. Correlation analyses were performed between critical thinking skills, *z*-scores of the area under the activity curve representing the oxy-Hb and deoxy-Hb concentrate values in the two ROIs, and the behavioral accuracy of the belief-bias syllogistic reasoning task. The *p*-values were all corrected using the FDR. Next, linear regressions were performed to examine the prediction of critical thinking by either the behavioral or hemodynamic indicators of the belief-bias syllogistic reasoning task.

## 3. Results

### 3.1. Behavioral Results

To examine differences in brain activity between individuals with varying levels of critical thinking, participants were divided into two groups based on their composite z-score of the two critical thinking measures (i.e., CCTT and CTHB). Participants who scored above the median score were classified as having high critical thinking, while those scoring below the median score were classified as having low critical thinking. This categorization into high and low critical thinking groups serves a practical purpose, as it simplifies the data analysis process. It also facilitates the generation of grand averages of brain activation for high and low critical thinking groups, enabling a clearer and more accessible comparison of neural patterns across groups. This method is commonly used in neuroimaging studies and enhances the interpretability and comparability of our results. The high critical thinking group consisted of 36 participants (26 males, *M*_age_ = 19.50, *SD* = 0.61), while the low critical thinking group consisted of 38 participants (24 males, *M*_age_ = 19.45, *SD* = 0.86). The results revealed a significant difference between the two groups in their composite z-score of critical thinking (high group: *M* = 0.71, *SD* = 0.32; low group: *M* = −0.68, *SD* = 0.72; *t* = −10.60, *p* < 0.01, Cohen’s *d* = 2.49). Furthermore, the high critical thinking group performed better on the belief-bias reasoning (accuracy of the experimental trials) compared to the low critical thinking group (high group: *M* = 0.91, *SD* = 0.08; low group: *M* = 0.75, *SD* = 0.19; *t* = −4.86, *p* < 0.01, Cohen’s *d* = 1.10). There was no significant difference in reaction times between conflict and non-conflict trials for all participants (conflict trials: *M* = 9326.19, *SD* = 2330.30; non-conflict trials: *M* = 9256.77, *SD* = 2349.39; *t* = 0.65, *p* < 0.05, Cohen’s *d* = 0.03). Additionally, significant correlations were observed between the accuracy of the belief-bias syllogistic reasoning task and performance on the CCTT (*r* = 0.47, *p* < 0.01) and CTHB (*r* = 0.44, *p* < 0.01) based on the total sample.

### 3.2. Brain Activity Between High and Low Critical Thinking Participants

**Figure 2:**
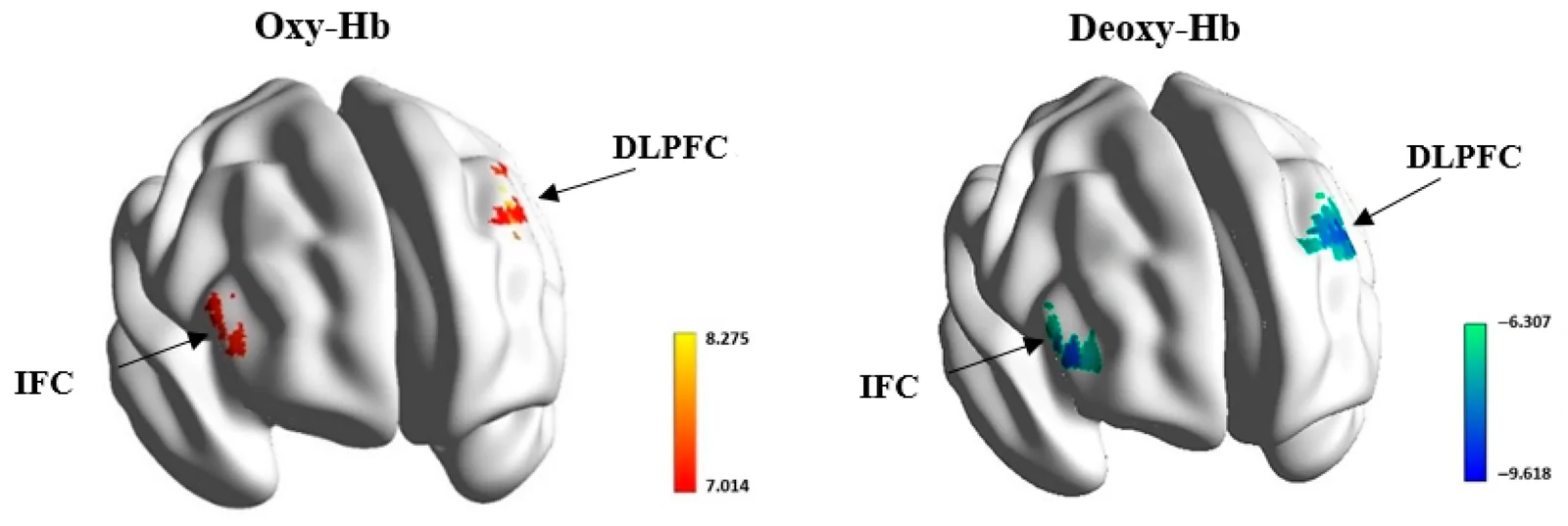
Activation maps of changes in oxy-Hb and deoxy-Hb values in completing the belief-bias reasoning task based on the total sample. Note. Activation maps were produced using the baseline-corrected block averaging method. *T*-values were calculated by areas under the activity curve for experimental trials and baseline trials. The activated brain areas include the left dorsolateral prefrontal cortex (DLPFC) and the opercular part of the right inferior frontal cortex (IFC). The color bar denotes the t-values. The activation map of oxy-Hb values was thresholded at the value of 7.014 to its maximum *t*-statistic (threshold: *p* < 0.001, FDR corrected). The activation map of deoxy-Hb values was thresholded at the value of −6.307 to its minimum *t*-statistic (threshold: *p* < 0.001, FDR corrected).

Figure 2 displays the activation maps of the oxy-Hb and deoxy-Hb concentrates when participants completed the experimental task. The activation maps depict the activated brain regions, including the opercular part of the right IFC and the left DLPFC. *T*-tests were performed on the oxy-Hb and deoxy-Hb concentrate values across the 53 channels for the contrast between experimental and baseline blocks. The resulting *p*-values were corrected using the FDR. Results showed that two channels in the left DLPFC (channel 5: *t* (73) = 7.01, *p* = 0.000, *p_corrected_* = 0.000, Cohen’s *d* = 0.82 and channel 6: *t* (73) = 8.28, *p* = 0.000, *p_corrected_* = 0.000, Cohen’s *d* = 0.96) and two channels in the opercular part of the right IFC (channel 46: *t* (73) = 7.06, *p* = 0.000, *p_corrected_* = 0.000, Cohen’s *d* = 0.82 and channel 51: *t* (73) = 7.48, *p* = 0.000, *p_corrected_* = 0.000, Cohen’s *d* = 0.87) exhibited significantly increased *t*-values in oxy-Hb than those in other channels. These higher *t*-values reflect greater changes in oxy-Hb, which corresponds to increased brain activation. Similarly, two channels in the left DLPFC showed significantly decreased *t*-values in deoxy-Hb (channel 5: *t* (73) = −6.31, *p* = 0.000, *p_corrected_* = 0.000, Cohen’s *d* = 0.73 and channel 6: *t* (73) = −9.62, *p* = 0.000, *p_corrected_* = 0.000, Cohen’s *d* = 1.12), along with two channels in the opercular part of the right IFC (channel 46: *t* (73) = −6.97, *p* = 0.000, *p_corrected_* = 0.000, Cohen’s *d* = 0.81 and channel 51: *t* (73) = −7.19, *p* = 0.000, *p_corrected_* = 0.000, Cohen’s *d* = 0.84). These lower *t*-values reflect greater changes in deoxy-Hb, corresponding to increased brain activation.

**Figure 3:**
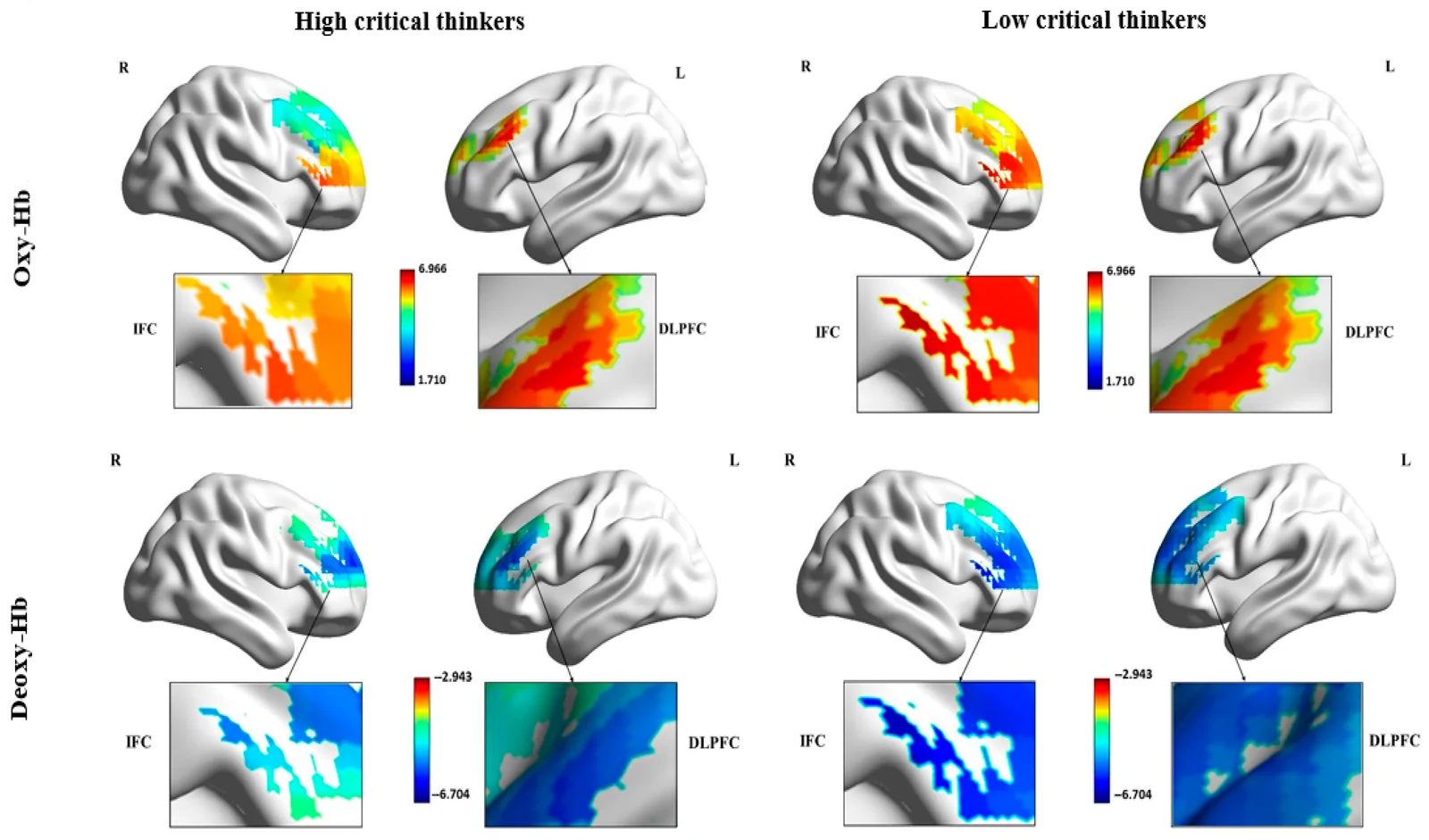
*T*-statistic maps of changes in oxy-Hb and deoxy-Hb values in completing the belief-bias syllogistic reasoning task for participants with high- and low levels of critical thinking. Note. Regions of interest associated with belief-bias syllogistic reasoning for participants with high levels of critical thinking (*N* = 36, *p* < 0.01, FDR corrected) and those with low levels of critical thinking (*N* = 38, *p* < 0.01, FDR corrected) include the left dorsolateral prefrontal cortex (DLPFC) and the opercular part of the right inferior frontal cortex (IFC). The resulting *p*-values were corrected using the false discovery rate method (FDR). The color bar denotes the *t*-values, with larger, higher *t*-values in oxy-Hb and lower *t*-values in deoxy-Hb representing greater changes in hemoglobin concentrations corresponding to greater brain activation.

**Figure 4:**
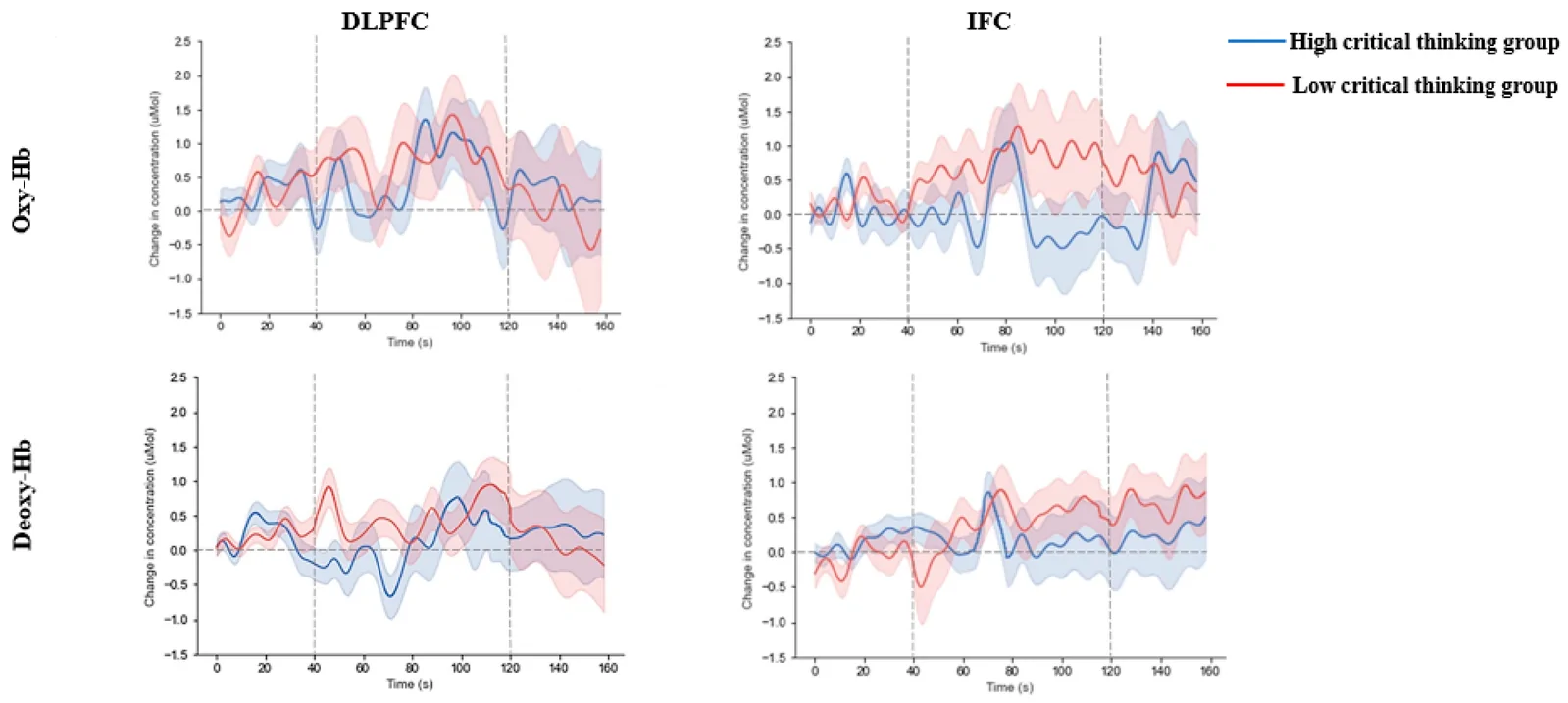
Waveform illustrations of oxy-Hb and deoxy-Hb concentration changes during belief-bias syllogistic reasoning for participants with high- and low levels of critical thinking. Note. Figure 4 illustrates the oxy- and deoxy-Hb concentration changes in the left dorsolateral prefrontal cortex (DLPFC) and the opercular part of the right inferior frontal cortex (IFC) associated with belief-bias reasoning (experimental time: 40–120 s) for participants with high- and low critical thinking levels. The standard errors of the mean were shown by the shaded bands.

Figure 3 illustrates the *t*-statistic maps reflecting the oxy-and deoxy-Hb concentrate values of each ROI across the two channels for high and low critical thinker groups. The figure indicates that low critical thinkers exhibit relatively higher activation in the ROIs, especially in the opercular part of the right IFC, compared to high critical thinkers (supplement E presents the *t*-test results). Figure 4 depicts the waveform maps illustrating fluctuations in oxy- and deoxy-Hb concentrations within the two ROIs for both high and low critical thinkers. Low critical thinkers exhibit larger changes in oxy- and deoxy-Hb concentrations in the opercular part of the right IFC than high critical thinkers (oxy-Hb: *t* = 3.09, *p* = 0.003, *p_corrected_* = 0.02, Cohen’s *d* = 0.72; deoxy-Hb: *t* = 2.87, *p* = 0.005, *p_corrected_* = 0.02, Cohen’s *d* = 0.67). No significant difference was found in the left DLPFC (see Table 1). Table 1 presents the mean concentration values of oxy-Hb and deoxy-Hb in the left DLPFC and the opercular part of the right IFC for both groups, as well as the *t*-test results. It should be noted that the areas under the activity curve were calculated as absolute values, with larger values representing greater changes in oxy- and deoxy-Hb concentrations. The findings suggest that the opercular part of the right IFC in low critical thinkers was more activated than that in high critical thinkers.

### 3.3. fNIRS and Behavioral Indicators of Belief-Bias Reasoning Predicting Critical Thinking

Table 2 presents correlations between critical thinking skills, the oxy-Hb and deoxy-Hb concentrate values of the two ROIs, and the behavioral accuracy of the belief-bias reasoning task based on the total sample. Accuracy of the reasoning task was moderately correlated with critical thinking skills (*r* = 0.51, *p* < 0.01). The oxy-Hb and deoxy-Hb concentrate values in both the opercular part of the right IFC and the left DLPFC were significantly correlated with critical thinking skills. Since both the oxy-Hb and deoxy-Hb concentrate values were calculated as areas under the activity curve, they exhibited similar correlations with critical thinking in terms of directions (positive or negative).

We then conducted linear regressions to examine the prediction of critical thinking skills by the behavioral or neural indicators of belief-bias reasoning. When the behavioral indicator, i.e., behavioral accuracy of the belief-bias reasoning task, was used as the predictor, the model explained 25% of the variance of critical thinking skills, *R*^2^ = 0.25, *F* = 24.36, *p* < 0.01. When the hemodynamic indicators, i.e., the oxy-Hb and deoxy-Hb concentrate values in the two ROIs, were used as predictors, they explained 33% of the variance of critical thinking skills, *R*^2^ = 0.33, *F* = 8.52, *p* < 0.01. The opercular part of the right IFC showed a negative association with critical thinking, as demonstrated by the significant effect of oxy-Hb (*β* = −0.39, *t* = −3.73, *p* < 0.01) and deoxy-Hb (*β* = −0.22, *t* = −2.05, *p* < 0.05) for critical thinking. The effect of oxy-Hb (*β* = −0.14, *t* = −1.35, *p* = 0.18) and deoxy-Hb (*β* = −0.11, *t* = −1.04, *p* = 0.30) in the left DLPFC were not significant.

To further reveal the contribution of hemodynamic changes to the prediction of critical thinking by belief-bias reasoning, we performed a hierarchical regression model with the hemodynamic variables on the first step and the behavioral variables on the second step. Critical thinking served as the dependent variable. The results showed that after controlling for the hemodynamic variables, the behavioral variables explained only 8% of the variance of critical thinking (*R*^2^ = 0.41, Δ*R*^2^ = 0.08, *F* = 9.60, *p* < 0.01). This suggests that hemodynamic activity accounted for approximately 64% (i.e., 0.64 = (0.25 − 0.08)/0.25) of the prediction from belief-bias reasoning to critical thinking.

## 4. Discussion

The current study was motivated by prior research suggesting that the ability to inhibit belief bias during reasoning is an essential aspect of critical thinking (Bensley 2023; Evans et al. 2022; West et al. 2008). Despite a growing body of research depicting the neural characteristics of belief-bias reasoning (e.g., Dubreuil-Vall et al. 2019; Goel and Dolan 2003; Tsujii and Watanabe 2009), there remained a gap in knowledge regarding the extent to which brain activity stimulated by belief-bias reasoning accounts for individual differences in critical thinking. To address this gap, the current study used fNIRS to examine brain activity during belief-bias syllogistic reasoning and to examine the relationship between this activity and critical thinking. Results show that the opercular part of the right IFC and left DLPFC were significantly activated when prior beliefs contradicted logical validity in completing the belief-bias syllogistic reasoning task. Crucially, those with high and low critical thinking displayed significant differences in the levels of oxy-Hb and deoxy-Hb concentration values in the opercular part of the right IFC. These hemodynamic changes explained a significant portion of the variance of critical thinking skills.

Our findings indicate that addressing conflicts between prior beliefs and logical validity during reasoning elicited significant activation in both the opercular part of the right IFC and the left DLPFC. From a neuroanatomical perspective, the opercular part of the right IFC has been proposed to be a component of the insula. The insula is well-known for counteracting biased information by facilitating top-down attentional control and inhibitory regulation over biased sensory input (Aron et al. 2014). Disruptions in the function of the right IFC have been associated with response control disorders (Aron et al. 2014). Neuroimaging research has consistently highlighted the crucial role of the right IFC in suppressing belief bias during reasoning (Goel and Dolan 2003; Tsujii et al. 2010). However, though the right IFC has been repeatedly revealed to be associated with belief-bias reasoning, prior research has not investigated the activation in other brain regions during belief-bias reasoning (Tsujii et al. 2010; Tsujii and Watanabe 2009). Our research expanded the scope of prior research to the entire prefrontal cortex and found that the left DLPFC was also recruited during belief-bias reasoning. This brain area has been identified as responsible for regulating working memory during conflict monitoring (Kim et al. 2014). Overall, these results suggest that belief-bias reasoning involves not just the activation of the right IFC but may be the result of multiple brain regions working in synergy.

Significant differences in oxy- and deoxy-Hb concentration values between high and low critical thinkers were observed in the right IFC, while no significant difference was found in the left DLPFC. As stated above, inhibiting belief bias during reasoning reflects the function of type 2 processing, heavily relying on cognitive resources such as working memory and executive functions (Evans and Stanovich 2013). This inhibitory function helps prevent individuals from confusing descriptions of the real world with those of imaginary scenarios, which is the process of “cognitive decoupling” as a core feature of type 2 processing (Evans and Stanovich 2013). Bensley (2020, 2023) also contends that a key function of critical thinking is the inhibition of default heuristic responses, including those influenced by belief bias. Importantly, individuals with low critical thinking abilities exhibited greater activation in the right IFC opercular region than those with high critical thinking abilities. Low critical thinkers are assumed to be more susceptible to belief bias and may need more attention and resources than higher critical thinkers. This result aligns with the neural efficiency hypothesis, which postulates that individuals with higher abilities tend to display lower brain activation than those with lower abilities when completing a cognitive task (Neubauer and Fink 2009). Lower activation suggests a more efficient allocation of cognitive resources toward problem-solving or decision-making (Neubauer and Fink 2009). While previous research testing this hypothesis has mainly focused on abilities closely tied to Spearman’s general abilities, our study extends the hypotheses by demonstrating that individuals with high critical thinking skills may use fewer attentional resources to resolve belief-logic conflicts during reasoning.

The results of the regression analyses have uncovered intriguing insights. It shows that both the oxy- and deoxy-Hb concentration values in the opercular region of the right IFC possessed a significant predictive capacity for critical thinking skills. Moreover, the hemodynamic changes in the opercular part of the right IFC and the left DLPFC accounted for a significant proportion of the association between accuracy in belief-bias reasoning and critical thinking. These findings not only provide empirical support for the previously postulated link between the suppression of belief-bias in reasoning and critical thinking, as posited by theoretical and empirical studies (Bensley 2023; Evans et al. 2022; Stanovich and Toplak 2019; Van Peppen et al. 2021; West et al. 2008), but also furnish a neural basis to elucidate this relationship. Specifically, individuals who efficiently allocate cognitive resources to resolve conflicts between beliefs and logic during reasoning demonstrate elevated levels of critical thinking skills. This result is significant as it justifies the inclusion of belief-based reasoning in most critical thinking assessment tools. Furthermore, these results align with findings from critical thinking interventions, highlighting the importance of providing explicit instructions to disentangle prior beliefs during reasoning to foster students’ critical thinking skills (e.g., Bensley 2023; Heijltjes et al. 2014; Van Peppen et al. 2021). However, it should be noted that the belief-based items in this study were developed by the researchers, so participants’ acceptance of these beliefs may have varied. This underscores the difference between externally imposed and personally relevant belief contradictions. Future research could better assess participants’ actual beliefs or tailor items to their personal relevance.

### 4.1. Research Implications

Results from this research have implications for the advancement of theoretical accounts of critical thinking. The dual-process account of reasoning suggests that the resolution of conflicts between belief and logic during reasoning is a crucial aspect of analytical thinking. However, this theory does not establish a direct link between belief-bias reasoning and critical thinking. Our study provides neural evidence for the behavioral link, and deepens the understanding of the brain functions for making impartial decisions during critical thinking. In addition, our results are in line with the metacognitive account of critical thinking. This theory provides a fresh perspective on the nature of critical thinking, with a particular focus on the role played by metacognitive monitoring and control processes (Halpern 2014). In this view, being mindful of one’s beliefs and recognizing and resolving any conflicts between beliefs and logical validity are considered metacognitive processes. Our study reveals a connection between critical thinking and the involvement of the left DLPFC and right IFC, which reflect attentional monitoring and inhibitory control for belief-bias reasoning. This extends the metacognitive account of critical thinking by incorporating a neural perspective.

The current study also has practical implications for educational interventions aimed at promoting critical thinking among students. The identification of the neural correlates of critical thinking has the potential to inform the development of more targeted and individualized educational interventions. By understanding the underlying neurophysiological processes involved in critical thinking, educators and researchers can tailor their approach to best suit the needs and strengths of each individual student. Additionally, previous assessments of critical thinking instruction programs have been criticized for relying on subjective methods, such as self-reported assessments (Abrami et al. 2015). By identifying neural signals that predict individual differences in critical thinking, this study sheds light on the potential for using objective neurophysiological markers as a means of evaluating the efficacy of these programs.

### 4.2. Limitations

This study employed a syllogistic reasoning paradigm to rigorously assess belief-bias inhibition while minimizing external confounds. Nevertheless, compared with classic multidimensional models, this neurocognitive approach may not fully capture the complexity of critical thinking as it manifests in real-world contexts, where reasoning is often embedded in ill-structured problems and influenced by personal, emotional, or contextual factors. Furthermore, the use of multiple-choice question formats, though widely adopted for their objectivity and standardization, may limit the accurate assessment of participants’ bias tendencies due to the possibility of guessing and the absence of open-ended responses that could reveal reasoning processes or metacognitive reflection. In addition, while this study focused on inhibitory control as one important pathway of critical thinking, it does not encompass other essential dimensions such as dispositions, epistemological understanding, and reflective judgment. Individuals may perform well on structured reasoning tasks yet fail to engage in critical thought when confronted with complex or belief-laden real-world issues. Integrating these aspects in future research would allow for a more comprehensive account of critical thinking. Finally, although the belief-laden conclusions used in the reasoning task were grounded in commonly held assumptions, participants’ actual beliefs were not directly measured. As a result, the extent of belief–logic conflict may have varied across individuals. Future studies could improve task validity by incorporating pre-task belief ratings to better align experimental stimuli with participants’ personal belief systems.

## 5. Conclusions

Our study examined the hemodynamic changes during belief-bias syllogistic reasoning and the association with critical thinking by means of fNIRS. The results indicate that participants with low critical thinking skills exhibited higher activation in the opercular part of the right IFC than those with high levels of critical thinking skills, whereas no such difference was found in the left DLPFC between high and low levels of participants. Moreover, the oxy- and deoxy-Hb concentration values in the regions of interest significantly contributed to the variance of critical thinking skills. Furthermore, we provide novel evidence that the hemodynamic changes, to a large extent, accounted for the behavioral association between belief-bias reasoning and critical thinking. These findings not only furnish a neural rationale for the association between belief-bias reasoning and critical thinking but also propel theoretical models of critical thinking by illuminating the brain’s processes involved in impartial reasoning and metacognition.

## Figures and Tables

**Table 1:** *T*-tests on oxy-Hb and deoxy-Hb values extracted from the regions of interest between participants with high- (*N* = 36) and low (*N* = 38) critical thinking skills.

	Levels of CT	*M*	*SD*	*t*	*p*	*p_corrected_*	Cohen’s *d*
Oxy-Hb							
Left DLPFC	Low CT group	57.10	37.93	0.74	0.46	0.96	0.17
	High CT group	50.69	36.97				
Opercular part of the right IFC	Low CT group	45.64	36.36	3.09	0.003	0.02	0.72
	High CT group	24.95	17.63				
Deoxy-Hb							
Left DLPFC	Low CT group	33.46	31.45	1.15	0.26	0.72	0.27
	High CT group	26.53	18.36				
Opercular part of the right IFC	Low CT group	32.24	24.05	2.87	0.005	0.02	0.67
	High CT group	18.68	15.45				

Note. The statistical models shown in this table were tested with the same degree of freedom (*df* = 72). Oxy-Hb, relative changes in oxyhemoglobin concentration; Deoxy-Hb, relative changes in deoxyhemoglobin concentration; DLPFC, dorsolateral prefrontal cortex; IFC, inferior frontal cortex; CT, critical thinking. The corrected *p*-values have been adjusted using the false discovery rate method.

**Table 2:** Correlations between accuracy of the belief-bias syllogistic reasoning task, the oxy- and deoxy-Hb values in the regions of interest and critical thinking skills (*N* = 74).

Measures	1	2	3	4	5	6
1. Accuracy of experimental trials of the belief-bias reasoning task	–					
2. Oxy-Hb in the left dorsolateral prefrontal cortex	−0.21	–				
3. Deoxy-Hb in the left dorsolateral prefrontal cortex	−0.26	0.13	–			
4. Oxy-Hb in the opercular part of the right inferior frontal cortex	−0.31 *	0.21	0.22	–		
5. Deoxy-Hb in the opercular part of the right inferior frontal cortex	−0.34 *	0.02	0.32 *	0.23	–	
6. Critical thinking skills	0.51 **	−0.24	−0.28 *	−0.49 **	−0.34 *	–

Note. Oxy-Hb, relative changes in oxyhemoglobin concentration. Deoxy-Hb, relative changes in deoxyhemoglobin concentration. Critical thinking skills were calculated by summing z-scores of the Chinese critical thinking test and the critical thinking skills test with heuristics and bias. ** *p* < 0.01, * *p* < 0.05. The resulting *p*-values have been corrected using the false discovery rate method.

## Data Availability

The original contributions presented in this study are included in the article/Supplementary Materials. Further inquiries can be directed to the corresponding author.

## Supplementary Materials

The following supporting information can be downloaded at: https://www.mdpi.com/article/10.3390/jintelligence13090106/s1, Figure S1: The Functional Near-infrared Spectroscopy Optode Arrangement and the Locations of 53 Channels; Table S1: Examples of Syllogisms Manipulating the Logical Validity and Believability of the Conclusions.
