# Supplementary material for: Neural Correlates of Belief-Bias Reasoning as Predictors of Critical Thinking: Evidence from an fNIRS Study
Source: J Intell. 2025 Aug 24;13(9):106. doi: 10.3390/jintelligence13090106 (PMC12471263; doi:10.3390/jintelligence13090106)
Supplement: Supplementary file 1 [file jintelligence-13-00106-s001.zip › jintelligence-3762640-supplementary.pdf]

Supplementary materials

A. Example materials of the belief-bias syllogistic reasoning task

Table S1

Examples of Syllogisms Manipulating the Logical Validity and Believability of the Conclusions.

Believability	Logical validity			
	Valid		Invalid	
Believable	All mammals are not birds	All mammals are not dogs	All pigeons are not mammals	All dogs are not mammals
	All dogs are mammals	All birds are mammals	All pigeons are birds	All mammals are birds
	All dogs are not birds	All dogs are not birds	All birds are not mammals	All dogs are not birds
Unbelievable	All mammals are not birds	All mammals are not pigeons	All birds are not dogs	All pigeons are not mammals
	All pigeons are mammals	All birds are mammals	All birds are mammals	All mammals are birds
	All pigeons are not birds	All pigeons are not birds	All mammals are not dogs	All pigeons are not birds

Note. Two types of syllogisms comprised of conflict trials and non-conflict trials were prepared. Each type included four logical forms. Blanks filled with grey color represent conflict trials, and those with white non-conflict trials.

B. locations of the probes in the Montreal Neurological Institute stereotaxic coordinate system

Figure S1

The Functional Near-infrared Spectroscopy Optode Arrangement and the Locations of 53 Channels.

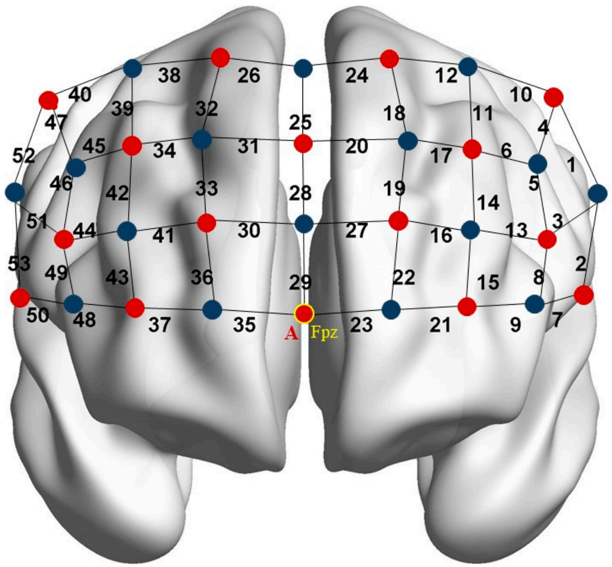

Note. Probes of the functional near-infrared spectroscopy optode were placed over the prefrontal regions. The red and blue circles represent the 16 emission probes and the 16 detector probes respectively. The digits denote 53 channels located in the frontal view using the BrainNet Viewer tool based on the Montreal Neurological Institute coordinate system. Optode A was located precisely at the midpoint of the frontal pole, as denoted by the 10-20 system notation for the position Fpz.

C. Example Items of the Chinese critical thinking test (CCTT)

We translated the example items measuring each sub-skill of critical thinking administered in the CCTT test from Chinese into English. Answers to the items were not provided due to copyright issues.

(1) Analysis

"Not all the birds can fly," expresses the same idea as:

A. None of the birds can fly.

B. Some birds cannot fly.

C. Someone who can fly is not a bird.

D. All birds cannot fly.

(2) Evaluation

"Petrol prices have soared due to the combined effects of the 2008 global financial crisis and the chaotic situation in the Middle East. In that same time the costs of several petroleum derivatives have also gone up sharply. These two facts establish that petrol is a petroleum derivative." The best evaluation of the speaker's reasoning is?

A. Good thinking, because petrol is a petroleum derivative.

B. Good thinking, but not all the facts are stated accurately.

C. Bad thinking. The cost of food has gone up in the same time, but that does not prove that petrol is food.

D. Bad thinking. One can draw no conclusions about petrol, given facts about petroleum derivatives.

(3) Deduction reasoning

A college student club has members from seven cities: Beijing, Shanghai, Guangzhou, Wuhan, Chongqing, Shenzhen, and Xi'an. The tutor must pick five students, each from a different city, to perform a play at the New Year celebration performance. Any combination of five students will do, except that if someone from Beijing is selected, no one from Chongqing should be selected. Also, if someone from Guangzhou is picked, someone

from Chongqing must be picked. And, if someone from Shanghai is selected, a member of Shenzhen must also be selected. Here are five possible combinations of students for the play. Which is the only combination that meets all the conditions?

A. Beijing, Shanghai, Guangzhou, Wuhan, Chongqing.

B. Shanghai, Guangzhou, Wuhan, Chongqing, Shenzhen.

C. Shanghai, Guangzhou, Wuhan, Shenzhen, Xi'an.

D. Beijing, Wuhan, Chongqing, Shenzhen, Xi'an.

E. Beijing, Shanghai, Guangzhou, Shenzhen, Xi'an.

(4) Inductive reasoning

Consider this argument: "Elephant L is smaller than elephant X. Elephant Y is smaller than elephant L, but elephant M is smaller than elephant Y. Therefore, elephant Y is smaller than elephant J." What information must be added to require that the conclusion be true, assuming all the premises are true?

A. Elephant L is bigger than J.

B. Elephant X is bigger than J.

C. Elephant J is bigger than L.

D. Elephant J is bigger than M.

(5) Inference reasoning

Consider this group of statements: "Shang Tang was the first Monarch of the Shang dynasty. Each of the Shang Monarchs drank wine and used bronze drinking vessels exclusively. Anyone who has used a bronze drinking vessel, even once, will have lead poisoning. Lead poisoning always manifests itself through insanity." Which of the following must be true if all of the above are true?

A. Those who suffer from insanity used the bronze drinking vessel at least once.

B. Whatever else, Monarch Shang Tang was certainly insane.

C. The exclusive use of bronze drinking vessels was the privilege of the Shang Monarchs.

D. Lead poisoning was common among the citizens of the Shang dynasty.

D. Example Items of the Critical thinking skills test with heuristics and biases (CTHB)

We translated the example items for each task category administered in the CTHB tests (√. correct answer) from Chinese into English. Explanations of the items and answers are also provided.

(1) Causal base-rate task

A trial tested 1,000 participants: 950 participants who buy their clothes at C&A and 50 participants who buy their clothes in exclusive clothes shops. Sacha was randomly selected out of those 1,000 participants. She is 36 years old, has a job as an asset manager at a bank, drives a Porsche, and lives in a Penthouse with her friend. How likely is it that Sacha buys her clothes in exclusive clothes shops?

A. more than 75%.

B. 50%.

C. 30%.

D. less than 10%. √

Explanation: This assignment requires participants not to confuse the logical validity of the conclusion with the believability of the conclusion, which probably seems unbelievable due to prior beliefs or real-world knowledge (Note that options A, B, and C demonstrate the tendency to base judgments on prior belief and to neglect the base-rate).

(2) Wason selection task

Each of the four cards below has an image on one side and a digit on the other side. The following rule is applied to the cards: If there is a heart on one side, then there is a 7 on the other side. Question: Which two cards do you think need to be turned over to verify the authenticity of this rule?

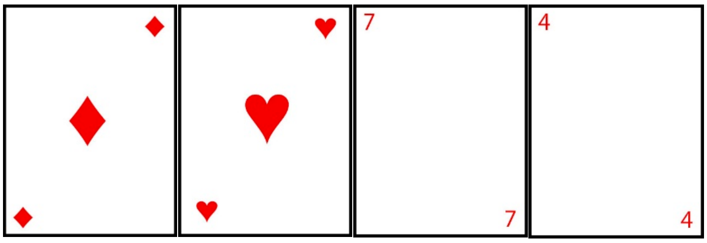

A. Heart, 7. B. Heart, 4. √ C. Diamond, 7. D. Diamond, 4.

Explanation: This assignment requires people to not only seek to confirm the rule but also look for falsification of the rule. By turning over the card with a heart, you can test whether the rule is violated: If there is no 7 on the other side, the rule is violated. The same for turning over the card with a 4: If that card has a heart on the other side, the rule is violated. Because if there is a heart on one side, there should be a 7 on the other side. People who choose other options than the combination of the card with a heart and the card with a 4, verify rules rather than falsify them, or demonstrate matching bias by selecting options explicitly mentioned in the conditional statement.

(3) Conjunction task

The Dutch national police has investigated crime in the major cities of the Netherlands. The city Rotterdam was part of the research and was selected by chance from the list of cities. Which of the following statements is most likely?

A. The Rotterdam police had to cut off staff and the number of street robberies in Rotterdam has increased.

B. The number of street robberies in Rotterdam has increased. √

Explanation: This assignment requires participants not to violate the conjunction rule which states that the probability of a conjunction cannot be more probable than one of its constituents.

(4) Covariation detection task

You are an entrepreneur and your company is on the brink of bankruptcy. Your neighbor tells you about Corporate Fixer: a company that specializes in solving business problems. “They do fantastic work”, he says, “the company of a good friend of mine became extremely successful after their help!” You visit their website and find out that the services of Corporate Fixer are quite pricey. You are prepared to pay the price, provided that you have a better chance of solving your business problems with their help than without any help. On an independent comparison website, you see that (a) 188 companies received help from corporate fixer and solved

their business problems, (b) 95 companies did not receive help and solved their problems, (c) 90 companies received help without solving their business problems, and (d) 25 companies did not receive help and did not solve their problems:

	Help from Corporate fixer	No help from Corporate fixer
Business problems solved	188	95
Business problem unsolved	90	25

Based on this information, would you commission from Corporate fixer or not?

A. Yes   B. No √

Explanation: The important thing to notice here is that one should evaluate the information given in a 2 × 2 contingency table equally and suppress the tendency to focus on the large number in the cell.

(5) Cognitive reflection task

If it takes 5 machines 5 minutes to make 5 widgets, how long would it take 100 machines to make 100 widgets?

A. 5 minutes. √ B. 100 minutes.

Explanation: This assignment requires people to override a prepotent response alternative that is incorrect and to engage in further reflection that leads to the correct response. “It takes 5 machines 5 minutes to make 5 widgets”, means every machine takes 5 minutes to produce 1 widget. So, it takes 5 minutes to make 100 widgets.

(6) Syllogistic reasoning task

You will find two premises below that you must assume are true. Indicate whether the conclusion follows logically from the given premises.

Premise 1: No lawyers are straightforward,

Premise 2: Some crooks are straightforward,

Conclusion: Some lawyers are no crooks.

Given that both premises are true,

A. the conclusion follows logically from the premises.

B. the conclusion does not follow logically from the premises. √

Explanation: The important thing to notice here is that one does not confuse the believability of the conclusion with the logical validity of the conclusion.

E. Comparing ROI Concentration Changes between Experimental Blocks and Baseline: T-Test Results for Participants with High- and Low Levels of Critical Thinking

For participants with high levels of critical thinking, *t*- tests revealed that two channels in the left LDPFC (channel 5: t (35) = 5.17, p = 0.000, $p_{corrected}$ = 0.000, Cohen's d = 0.81 and channel 6: t (35) = 4.51, p = 0.000, $p_{corrected}$ = 0.000, Cohen's d = 0.74) and two channels in the opercular part of the right IFC (channel 46: t (35) = 5.06, p = 0.000, $p_{corrected}$ = 0.000, Cohen's d = 0.83 and channel 51: t (35) = 3.67, p = 0.001, $p_{corrected}$ = 0.01, Cohen's d = 0.60) with higher *t*- values in oxy-Hb than other channels. Likewise, for deoxy-Hb, channel 5 (t (35) = -4.88, p = 0.000, $p_{corrected}$ = 0.000, Cohen's d = 0.80) and channel 6 (t (35) = -3.84, p = 0.000, $p_{corrected}$ = 0.000, Cohen's d = 0.63), as well as two channels in the opercular part of the right IFC (channel 46: t (35) = -4.58, p = 0.000, $p_{corrected}$= 0.000, Cohen's d = 0.75; channel 51: t (35) = -3.2, p = 0.000, $p_{corrected}$ = 0.000, Cohen's d = 0.53), showed significantly lower *t*- values.

For participants with low levels of critical thinking, two channels in the left LDPFC (channel 5: t (37) = 6.97, p = 0.000, $p_{corrected}$ = 0.000, Cohen's d = 1.15; channel 6: t (37) = 5.09, p = 0.000, $p_{corrected}$ = 0.000, Cohen's d = 0.84) and two channels in the opercular part of the right IFC (channel 46: t (37) = 4.30, p = 0.000, $p_{corrected}$ = 0.000, Cohen's d = 0.71; channel 51: t (37) = 6.68, p = 0.000, $p_{corrected}$ = 0.000, Cohen's d = 1.10) exhibited significant difference between experimental blocks and baseline, with higher *t*- values in oxy-Hb than other channels. Similarly, for deoxy-Hb, channel 5 (t (37) = -6.70, p = 0.000, $p_{corrected}$ = 0.000, Cohen's d = 1.10) and channel 6 (t (37) = -4.63, p = 0.000, $p_{corrected}$ = 0.000, Cohen's d = 0.76), along with two channels in the opercular part of the right IFC (channel 46: t (37) = -4.07, p = 0.000, $p_{corrected}$ = 0.000, Cohen's d = 0.67; channel 51: t (37) = -6.52, p = 0.000, $p_{corrected}$ = 0.000, Cohen's d = 1.01), showed significant difference between experimental blocks and baseline, with lower *t*- values in deoxy-Hb.
